# Liver-targeting MRI contrast agent based on galactose functionalized o-carboxymethyl chitosan

**DOI:** 10.3389/fbioe.2023.1134665

**Published:** 2023-05-22

**Authors:** Li Xu, Zhanying Ren, Guolin Li, Danni Xu, Jiaqian Miao, Jingxuan Ju, Xuan Mo, Xianghui Wang, Hongping Deng, Min Xu

**Affiliations:** ^1^ Shanghai Frontiers Science Center of TCM Chemical Biology, Institute of Interdisciplinary Integrative Medicine Research, Shanghai University of Traditional Chinese Medicine, Shanghai, China; ^2^ Shanghai Key Laboratory of Magnetic Resonance, School of Physics and Electronic Science, East China Normal University, Shanghai, China; ^3^ Southern University of Science and Technology, Shenzhen, China; ^4^ Department of Stomatology, Shanghai 8th People’s Hospital, Shanghai, China

**Keywords:** gadolinium (Gd)-based MRI contrast agents, liver-targeting, imaging contrast, galactose functionized o-carboxymethyl chitosan, biocompatibility

## Abstract

Commercial gadolinium (Gd)-based contrast agents (GBCAs) play important role in clinical diagnostic of hepatocellular carcinoma, but their diagnostic efficacy remained improved. As small molecules, the imaging contrast and window of GBCAs is limited by low liver targeting and retention. Herein, we developed a liver-targeting gadolinium (Ⅲ) chelated macromolecular MRI contrast agent based on galactose functionalized o-carboxymethyl chitosan, namely, CS-Ga-(Gd-DTPA)_n_, to improve hepatocyte uptake and liver retention. Compared to Gd-DTPA and non-specific macromolecular agent CS-(Gd-DTPA)_n_, CS-Ga-(Gd-DTPA)_n_ showed higher hepatocyte uptake, excellent cell and blood biocompatibility *in vitro.* Furthermore, CS-Ga-(Gd-DTPA)_n_ also exhibited higher relaxivity *in vitro,* prolonged retention and better T1-weighted signal enhancement in liver. At 10 days post-injection of CS-Ga-(Gd-DTPA)_n_ at a dose of 0.03 mM Gd/Kg, Gd had a little accumulation in liver with no liver function damage. The good performance of CS-Ga-(Gd-DTPA)_n_ gives great confidence in developing liver-specifc MRI contrast agents for clinical translation.

## 1 Introduction

Liver cancers, including hepatocellular carcinoma, hepatic lymphoma, hepato-cellular adenoma, have become highly lethal cancers worldwide (Forner et al., 2018; Villanueva, 2019; Sung et al., 2021; Ganesan and Kulik, 2023). Most patients have already been in the advanced stage of hepatocarcinogenesis once confirmed (Maluccio and Covey, 2012; Anwanwan et al., 2020). Thus, accurately detecting liver cancer lesions in the early stage of hepatocarcinogenesis is urgently needed for clinical diagnostic. Liver magnetic resonance imaging (MRI) is a non-invasive approach, which can provide valuable pathophysiological state information and clinically recommended for liver disease diagnosis (Choi et al., 2014; Choi et al., 2014; Vilgrain et al., 2016). Choosing appropriate liver contrast agents is of great importance to obtain high-resolution liver tissue images (Welle et al., 2020).

Currently, there are eight commercial gadolinium (Gd)-based contrast agents (GBCAs) are available for liver MRI diagnosis: Gadopentetate dimeglumine (Gd-DTPA), Gadodiamide (Gd-DTPA-BMA), Gadoteridol (Gd-HP-DO3A), Gadoversetamide (Gd-DTPA-BMEA), Gadoterate meglumine (Gd-DOTA), Gadobutrol (Gd-BT-DO3A), Gadobenate dimeglumine (Gd-BOPTA), Gadoxetic acid (Gd-EOB-DTPA) (Hope et al., 2015; Kim et al., 2015; Okada et al., 2016; Glockner et al., 2018; Li and Meade, 2019; Kang et al., 2020; Kim, 2020; Fatima et al., 2021). Gd-EOB-DTPA and Gd-BOPTA are hepatobiliary-specific agents which can be taken up by hepatocytes and excreted from bile, while others are extracellular fluid agents which cannot be actively uptake by cells, only distribute in extracellular fuid and excrete from kidneys (Faletti et al., 2015). Generally, extracellular fluid agents can be used in most liver lesions detection. In contrast, hepatobiliary-specific MRI agents have hepatobiliary phase and are preferred in assess for focal nodular hyperplasia diagnosis, which refers live metastatic lesions, a uncertain lesion identified by ECA exam, hepatic function or abnormalities within the bile ducts. However, there still exists limitations for hepatobiliary-specific agents (Goodwin et al., 2011; Lee et al., 2012; McInnes et al., 2015; Besa et al., 2017). First, Gd-EOB-DTPA and Gd-BOPTA are small molecule agents with short half life which reduce its bioavailability and fail to use in some diagnosis which need long imaging window. Second, gadolinium residues cause safety risks by repeating dose or high dose (Li and Meade, 2019; Oluwasola et al., 2022). In addtion, GBCAs is associated with nephrogenic systemic fibrosis (NSF) (McDonald and McDonald, 2020). Medicines and Healthcare products Regulatory Agency (MHRA) has limited the use of Gd-DTPA, Gd-DTPA-BMA, and Gd-DTPA-BMEA in patients with renal dysfunction since 2007, because they could raise the risk of nephrogenic systemic fibrosis (NSF). NSF is a rare disease and occurs only in patients with renal dysfunction, which can lead to serious physical disability such as skin and connective tissue fibrosis, joint movement disorder and other organs dysfunction, even can over serious patients’ life.

**SCHEME 1:**
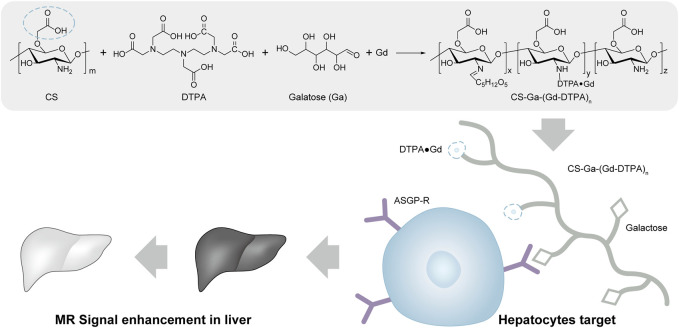
Synthesis of CS-Ga-(Gd-DTPA)_n_ and its application in liver MRI diagnosis, the blue circle denotes the carboxymethyl group on the chemical structure of chitosan.

To address these issues, it is urgent to develop liver specific GBCAs with higher relaxivity, better chemical stability and biocompatibility (Lancelot et al., 2020; Zhang et al., 2022). Thus, constructing liver targeted macromolecular MRI contrast agent by grafting small MRI contrast agent and liver targeting molecule on biocompatible and biodegradable polymer carriers is highly attentioned. Compared to small agents, the macromolecular MRI contrast agents have higher relaxivity, better chemical stability, prolonged circulation time and are flexible to achieve liver targeting by integrating liver targeting molecule on its structure. Chitosan is a biocompatible polysaccharide that has been widely used in drug delivery (Dash et al., 2011; Kyzas and Bikiaris, 2015; Muxika et al., 2017; Abd EI-Hack et al., 2020; Lang et al., 2020; Zheng et al., 2020; Lima et al., 2021; Frigaard et al., 2022). Carboxymethyl chitosan is a water soluble derivative of chitosan with a variety of carboxyl groups, which brings negative charge for long time circulation and functional ligand for modification of target groups and MRI contrast agents (Hata and Ishii, 1998; Geraldes and Peters, 2022). Herein, we develop a liver-targeting MRI contrast agent CS-Ga-(Gd-DTPA)_n_. We functionalized carboxymethyl chitosan with galactose (Ga), which can be specifically recognized by asialoglycoprotein receptor (ASGP-R) on the surface of mammalian hepatocytes and internalized via a receptor-mediated endocytosis process (Hata and Ishii, 1998; Mukthavaram et al., 2009). DTPA was also grafted onto the carboxymethyl chitosan to improve the chelation stability of Gd. In the meantime, non liver-specific agent CS-(Gd-DTPA)_n_ is synthesized as a control agent. Compared to small Gd-DTPA, liver-specific macromolecular CS-Ga-(Gd-DTPA)_n_ has higher relaxivity, longer retention time and high accumulation in liver, which can provide better MR contrast enhancement, longer effective imaging window with lower dose, and reduce its safety risk (Scheme 1).

## 2 Materials and methods

### 2.1 Materials

Carboxymethyl chitosan (CS, DS ≥ 80%, average molecular weight 100 kD), D-galactose and DTPA were purchased from Sigma. Dimeglnmine gadopentetate (Gd-DTPA) was purchased from Bayer Schering Pharma (Cambridge, UK). Cell Counting Kit-8 (CCK-8) was purchased from DojinDo laboratory. BCA Protein Quantification Kit was purchased from Beyotime Institute of Biotechnology (Shanghai, China). HUVEC, HepG2, HepaRG, L929 cell lines were purchased from ATCC. Sprague-Dawley rats were purchased from specific pathogen free (SPF) laboratory animal center in East China Normal University. Hematoxylin-eosin was purchased from Bioeasy Biological Co. Ltd (Shanghai, China). Anticoagulant citrate dextrose (ACD) human blood was provided by healthy donors.

### 2.2 Cell culture

Human umbilical vein endothelial cells (HUVEC) were cultured in F-12 K medium supplemented with 1% penicillin, 1% streptomycin and 10% fetal bovine serum. HepG2 were cultured in dulbecco’s modified eagle medium (DMEM) containing 1% penicillin, 1% streptomycin and 12% fetal bovine serum. L929 cells were cultured in DMEM medium containing 1% penicillin, 1% streptomycin and 10% fetal bovine serum. HepaRG cells were cultured in William’s E medium containing 1% penicillin, 1% streptomycin, 1% Glutamax, 1% insulin-transferrin-selenium, 10 μM dexamethasone and 10% FBS. The cells were cultured at 37°C, 5% CO_2_ in a humidified incubator and passaged when cells were 80% confluence.

### 2.3 Synthesis and characterization of CS-DTPA and CS-Ga-DTPA

0.5 g CS was dissolved in 40 mL deionized water, then 17 g DTPA was added. Stirred the solution and dropped 5 M NaOH until the DTPA was completely dissolved. The pH of above solution was adjusted to 5. 1-(3-Dimethylaminopropyl)-3-ethylcarbodiimide and 1-Hydroxybenzotri-zole (EDC·HCl) was added in the solution as condensation agents, and kept stirring in 110°C oil bath for 24 h. Finally, the reaction solution was dialyzed in deionized water and frozen drying to yield solid samples CS-DTPA. For CS-Ga-DTPA preparation, above reaction solution was adjusted to pH = 7-8 with 0.2 M disodium hydrogen phosphate solution, then appropriate amount of galactose was added. Then the reaction solution was kept stirring for 24 h under 110°C oil bath. The reaction solution was dialysis against deionized water and then frozen drying to yield solid samples CS-Ga-DTPA. ^1^H NMR and ^13^C NMR spectrum were used to calculate the grafting ratio of DTPA and galactose on CS (Bruker Avance-500 spectrometer). The average hydrodynamic diameter of CS-DTPA and CS-Ga-DTPA were detected by dynamic light scattering (DLS) on Zetasizer Nano S.

### 2.4 Synthesis and characterization of CS-(Gd-DTPA)_n_ and CS-Ga-(Gd-DTPA)_n_

0.1 M Gd^3+^ was added slowly to CS-DTPA and CS-Ga-DTPA aqueous solution respectively, following by stirring for 1 h at room temperature. Both reaction solution was dialysis against deionized water for 2 days, and then frozen drying to yield solid samples CS-(Gd-DTPA)_n_ and CS-Ga-(Gd-DTPA)_n_. The Gd content is detected on inductively coupled plasma spectrometry (ICP).

### 2.5 *In vitro* relaxivity

Prepared 10 mL H_2_O and 10 mL CS-(Gd-DTPA)_n_, CS-Ga-(Gd-DTPA)_n_ and Gd-DTPA aqueous solution with different concentrations (0.05 mM Gd/L, 0.1 mM Gd/L, 0.2 mM Gd/L) respectively. The Siemens Trio 3 T Scanner was used to collect T_1_-weighted images of these solutions, with an inversion recovery prepared T_1_-weighting spin-echo pulse sequence.

### 2.6 Cell uptake

To evaluate the hepatocytes target of CS-Ga-DTPA, CS-Ga-DTPA was labeled with TRITC and incubated with different cells: human hepatic cancer cell line HepG2, human hepatic cell line HepaRG, and mouse fibroblast cell line L929. 0.5 μM CS-Ga-DTPA-TRITC was added in the medium of HepG2, HepaRG and L929 cells and co-cultured for 1 h, respectively. The cells were washed, harvested and analyzed on flow cytometer.

### 2.7 *In vitro* biocompatibility

To evaluate the cytotoxicity of CS-DTPA, CS-Ga-DTPA, CS-Ga-(Gd-DTPA)_n_ and CS-(Gd-DTPA)_n_, CCK-8 assay on HepaRG and HUVEC cells were conducted. Cells were seeded in 96-well plates at a density of 8×10^3^ cells per well. After 24 h, cells were co-cultured with CS-DTPA (0.05 mg/mL), CS-Ga-DTPA (0.05 mg/mL), CS-(Gd-DTPA)_n_, CS-Ga-(Gd-DTPA)_n_ and Gd-DTPA at serial final concentrations (0.001 mM Gd/L-0.25 mM Gd/L) for preset time. To evaluate the hemolysis of CS-Ga-(Gd-DTPA)_n_, anticoagulant citrate dextrose (ACD) human blood was used to test the hemolysis ratio. 4 mL blood was diluted in 5 mL PBS. 5 μL samples (CS-(Gd-DTPA)_n_, CS-Ga-(Gd-DTPA)_n_ and Gd-DTPA) with different concentrations were added in 45 μL diluted blood in treated groups. Then 5 μL PBS was added in 45 μL diluted blood in negative control groups (n = 3), while 5 μL ultrapure water was added in positive control groups (n = 3). The solutions in all groups were incubated at 37 °C for 1 h, then centrifuged at 3,000 rpm for 5 min. The optical density of the supernatant was measured at 450 nm on a microplate reader. Hemolysis ratio was calculated via the formula: HR= (A-AN)/(AP-AN) ×100%, Where HR is the hemolysis ratio (%), A is absorbency of the samples (%), AP is absorbency of the positive controls (%), and AN is absorbency of the negative controls (%).

### 2.8 *In vivo* MRI

*In vivo* T_1_–weighted MRI images were acquired on a Siemens Trio 3T Scanner with Siemens 3T Animal Coil before and at different time points after injection of CS-(Gd-DTPA)_n_, CS-Ga-(Gd-DTPA)_n_ and Gd-DTPA. A group of 5 male Sprague-Dawley (SD) rats (180–200 g) were used to study the signal enhancement of CS-(Gd-DTPA)_n_, CS-Ga-(Gd-DTPA)_n_ and Gd-DTPA *in vivo*. All animal operations were conducted in accordance with the Chinese guidelines on laboratory animals’ use and care. Rats were anesthetized by intraperitoneal injection of 1% pentobarbital sodium (1 g in 100 mL physiological saline)at a dose of 0.01 g/g body weight. CS-(Gd-DTPA)_n_, CS-Ga-(Gd-DTPA)_n_ and Gd-DTPA were administrated via tail vein at a dose of 0.03 mM Gd/Kg, respectively. MR images were acquired before and at 0 min, 5 min, 15 min, 1 h, 3 h, 6 h and 12 h post injection. The measurement parameters were as follows: slice thickness (SL) = 2 mm, repetition time (TR) = 1,120 ms, echo time (TE) = 24 ms, filed of view (FOV) = 60 × 60 cm^2^, matrix size = 512 × 512. *In vivo* contrast enhancement in the liver was analyzed by Image J software.

### 2.9 *In vivo* biosafety

To evaluate the biosafety of CS-Ga-(Gd-DTPA)_n_, tissue biodistribution of Gd, organs (heart, liver, lung, spleen and kidneys) histopathological analysis, biochemical analysis of blood and serum level of inflammation factor were conducted. SD rats (5 rats per group, 180–200 g) were administrated CS-(Gd-DTPA)_n_ and CS-Ga-(Gd-DTPA)_n_ via tail vein at equal dose of 0.03 mM Gd/Kg. The rats were euthanized and dissected at 10 days post injection. The blood was collected and coagulated for 1 h at room temperature. The supernatant was centrifuged at 3,000 rpm for 10 min to obtain serum for quantification of interleukin 1β (IL-1β), tumor necrosis factor (TNF-α), aspartate aminotransferase (AST), alanine aminotransferase (ALT), alkaline phosphatase (ALP), blood urea nitrogen (BUN) and creatinine (CREA). The blood was washed away by transcardial perfusion with physiological saline, and then organs (heart, lung, spleen, kidneys and liver) were removed and weighed. The organs were cut into1-2 mm^2^ pieces and digested in mixed acids (nitric acid: perchloric acid = 4:1) for 48 h. The content of gadolinium in organs was determined by ICP-AES. A little pieces of tissues were cut from liver, fixed by formaldehyde, and then dehydrated and embedded in paraffin. About 5 μm transverse sections were cut and stained with hematoxylin-eosin (HE) for histopathological analysis.

### 2.10 Statistical analysis

Statistical data are acquired from at least three times experiments and showed as mean ± standard deviation (SD). All quantitative data were analyzed by two-tailed Student’s t test. *p* > 0.05 (NS) denotes there is no statistically significant. *p* < 0.05 (*), *p* < 0.01 (**), *p* < 0.001 (***), were defined as statistically significant.

## 3 Results and discussion

### 3.1 Characterization of CS-Ga-DTPA and CS-DTPA

**FIGURE 1:**
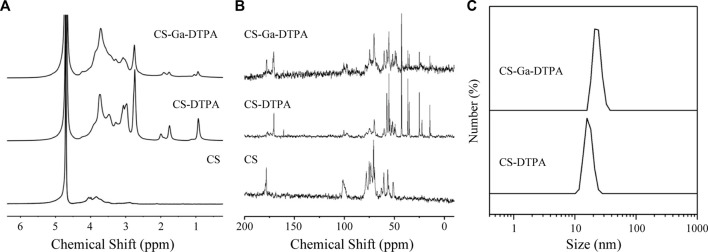
Characterizations of CS-DTPA and CS-Ga-DTPA. **(A)** ^1^H NMR spectrum of CS, CS-DTPA and CS-Ga-DTPA in D_2_O. **(B)** ^13^C NMR spectrum of CS, CS-DTPA and CS-Ga-DTPA in D_2_O. **(C)** Hydrodynamic diameter of CS-DTPA and CS-DTPA.

Chitosan is a biocompatible polysaccharide that has been widely used in biomedical sciences. Carboxymethyl chitosan is a derivative of chitosan with a variety of carboxyl groups, which brings negative charge for long time circulation and functional ligand for modification of contrast agents. We intended to develop a liver targeted MRI contrast agents by labeling carboxymethyl chitosan with Gd-DTPA and galactose (Ga). As shown in Figures 1A, B, both ^1^H and ^13^C NMR illustrated the successful preparation of CS-DTPA and CS-Ga-DTPA with grafting ratio of 10.2% and 25.1% for DTPA and Ga respectively. CS-DTPA and CS-Ga-DTPA were very soluble in water and did not self-assemble into nanoparticles. Thus, the hydrodiameter of CS-DTPA was just around 15.7 nm, which further increased to around 21 nm after conjugating with Ga (Figure 1C). Details for the synthesis of CS-DTPA and CS-Ga-DTPA were provided in the experimental section.

### 3.2 Hepatocytes target

**FIGURE 2:**
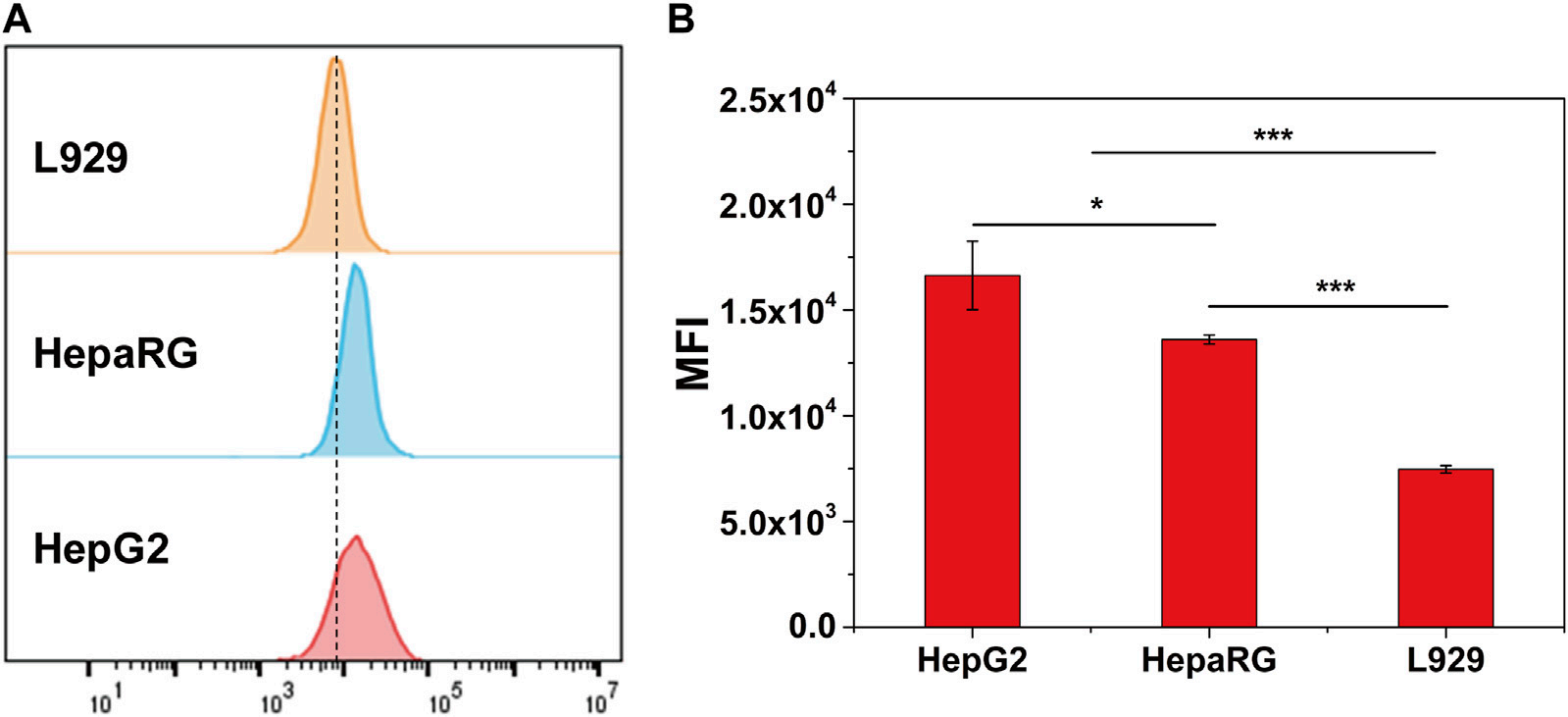
Cellular uptake behavior of CS-Ga-DTPA. **(A)** The cellular uptake of TRITC labeled CS-Ga-DTPA after co-cultured with HepG2, HepaRG and L929 cells for 1 h respectively. **(B)** Mean fluorescence intensity of HepG2, HepaRG and L929 cells.

To evaluate the hepatocytes target of CS-Ga-DTPA_,_ TRITC cnjugated CS-Ga-DTPA was co-cultured with three types cell lines: human hepatic cancer cell HepG2, human hepatic cell HepaRG, and mouse fibroblast cell L929 for 1 h, respectively. The flow cytometer analysis showed both HepG2 and HepaRG have high mean fluorescence intensity in comparison with L929 (Figure 2), demonstrating that CS-Ga-DTPA is hepatocytes target.

### 3.3 *In vitro* biocompatibility

**FIGURE 3:**
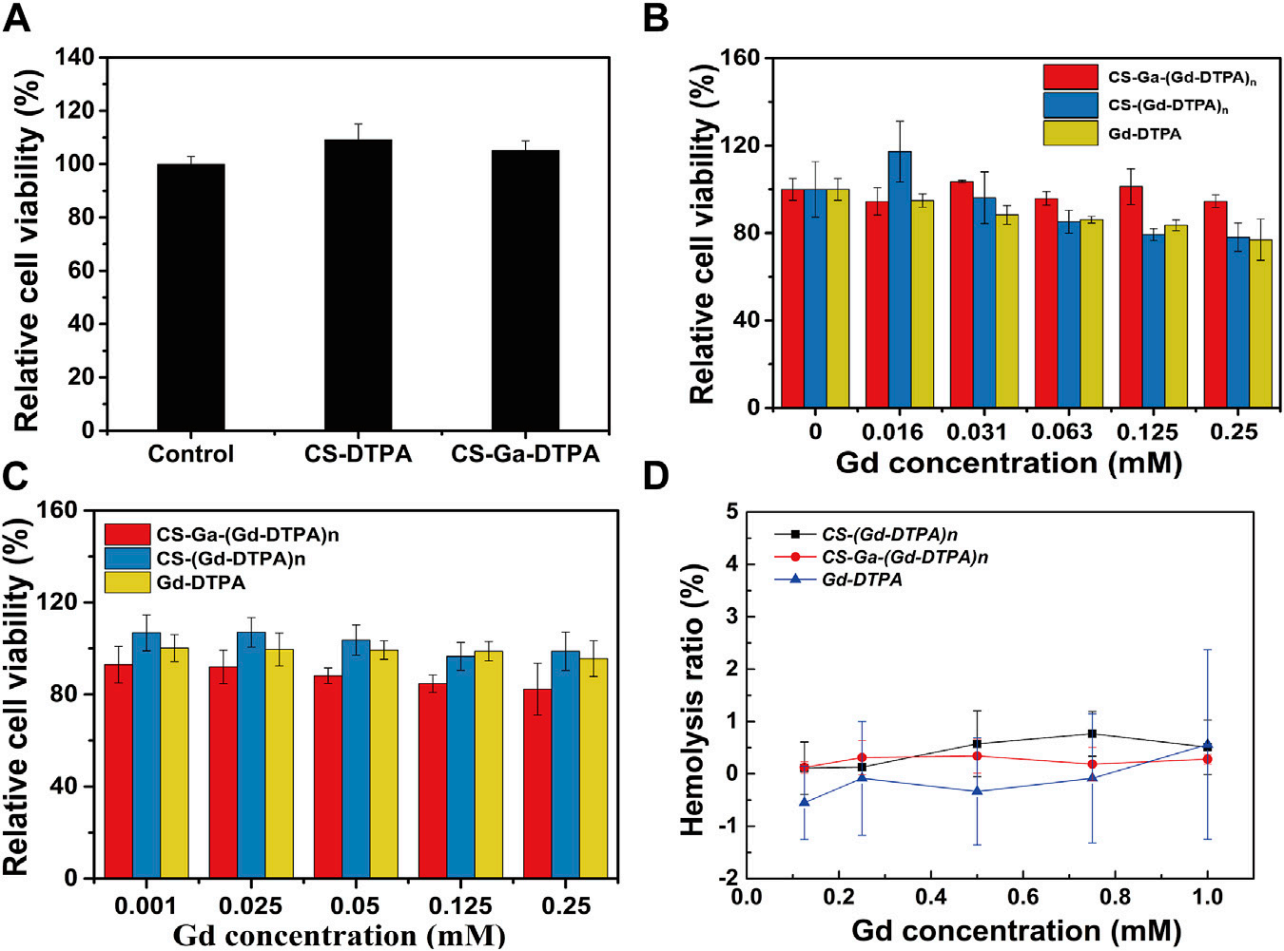
*In vitro* biocompatibility of CS-Ga-(Gd-DTPA)_n_. **(A)** The relative cell viability of HepaRG cells after co-cultured with CS-DTPA (0.05 mg/mL) and CS-Ga-DTPA (0.05 mg/mL) for 72 h measured by CCK-8 assay. **(B)** The relative cell viability of HepaRG cells after co-cultured with CS-Ga-(Gd-DTPA)_n_, CS-(Gd-DTPA)_n_ and Gd-DTPA for 72 h measured by CCK-8 assay. **(C)** The relative cell viability of HUVEC cells after co-cultured with CS-Ga-(Gd-DTPA)_n_, CS-(Gd-DTPA)_n_ and Gd-DTPA for 24 h measured by CCK-8 assay. **(D)** Hemolysis ratio of human blood treated by CS-Ga-(Gd-DTPA)_n_, CS-(Gd-DTPA)_n_ and Gd-DTPA at different concentrations of Gd.

To evaluate the *in vitro* cytotoxicity of CS-DTPA, CS-Ga-DTPA, CS-Ga-(Gd-DTPA)_n_ and CS-(Gd-DTPA)_n_, human hepatocytes (HepaRG) and vascular endothelial cells (HUVEC) cell lines were used. As CS-Ga-(Gd-DTPA)_n_ is designed to target liver to prolong liver retention for better liver MRI imaging, the treatment time of CS-DTPA, CS-Ga-DTPA, CS-Ga-(Gd-DTPA)_n_ and CS-(Gd-DTPA)_n_ on hepatocytes was 72 h, longer than vascular endothelial cells (24 h). Both CS-DTPA (0.05 mg/mL) and CS-Ga-DTPA (0.05 mg/mL) have no toxicity on HepaRG cells after co-cultured for 72 h, of which dosage is equal to the content of carrier materials at the maximum dose of CS-Ga-(Gd-DTPA)_n_ and CS-(Gd-DTPA)_n_ (0.25 mM Gd/L) (Figure 3A). After co-cultured with serial concentrations of CS-Ga-(Gd-DTPA)_n_ for 72 h, HepaRG cells showed higher cell viability than cells co-cultured with equal Gd concentration of CS-(Gd-DTPA)_n_ and Gd-DTPA, especially at high concentrations (0.063 mM Gd/L-0.25 mM Gd/L) (Figure 3B). After co-cultured with serial concentrations of CS-Ga-(Gd-DTPA)_n_ for 24 h, the relative cell viability of HUVEC cells were all over 80%, even at the concentration as high as 0.25 mM Gd/L, far greater than the dose used in *in vivo* MRI experiments (0.03 mM Gd/kg) (Figure 3C). These data demonstrated that CS-Ga-(Gd-DTPA)_n_ has no cytotoxicity at its effective dose *in vitro*.

The blood compatibility of CS-Ga-(Gd-DTPA)_n_ was evaluated by hemolysis rate. No hemolysis cases was observed in blood samples treated by CS-Ga-(Gd-DTPA)_n_, CS-(Gd-DTPA)_n_ and Gd-DTPA. The hemolysis rate of three agents at all treated doses were all around zero (Figure 3D) and far less than 5% which was the national safety standards for drugs. These data primarily demonstrated that CS-Ga-(Gd-DTPA)_n_ and CS-(Gd-DTPA)_n_ showed as good blood compatibility as commercial agent Gd-DTPA.

### 3.4 *In vitro* relaxivity

**FIGURE 4:**
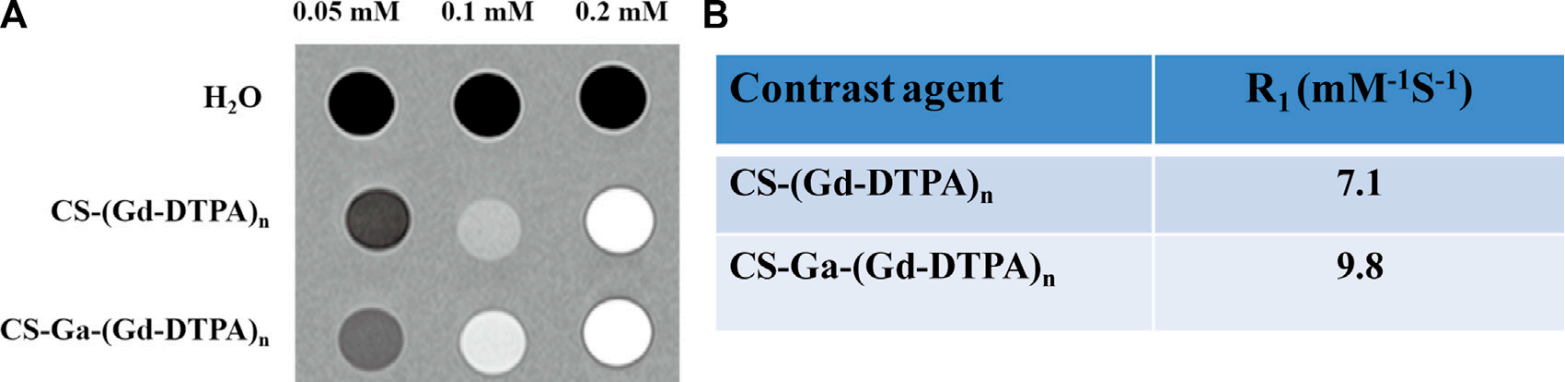
**(A)** T_1_-weighted MR images of H_2_O, CS-(Gd-DTPA)_n_ and CS-Ga-(Gd-DTPA)_n_. **(B)** The longitudinal relaxivity (R_1_) of CS-(Gd-DTPA)_n_ and CS-Ga-(Gd-DTPA)_n_.

The *in vitro* relaxivity of CS-Ga-(Gd-DTPA)_n_ and CS-(Gd-DTPA)_n_ were measured on a Siemens Trio 3T Scanner. Based on the T1-weighted images (Figure 4A), the relaxivity of CS-Ga-(Gd-DTPA)_n_ was calculated as 9.8 mM^‐1^S^−1^, which is 2.2 times higher than Gd-DTPA (4.3 mM^‐1^S^−1^) measured in our previous study (Wang et al., 2019) and 1.38 times higher than CS-(Gd-DTPA)_n_ (7.1 mM^‐1^S^−1^) (Figure 4B).

### 3.5 *In vivo* MRI

**FIGURE 5:**
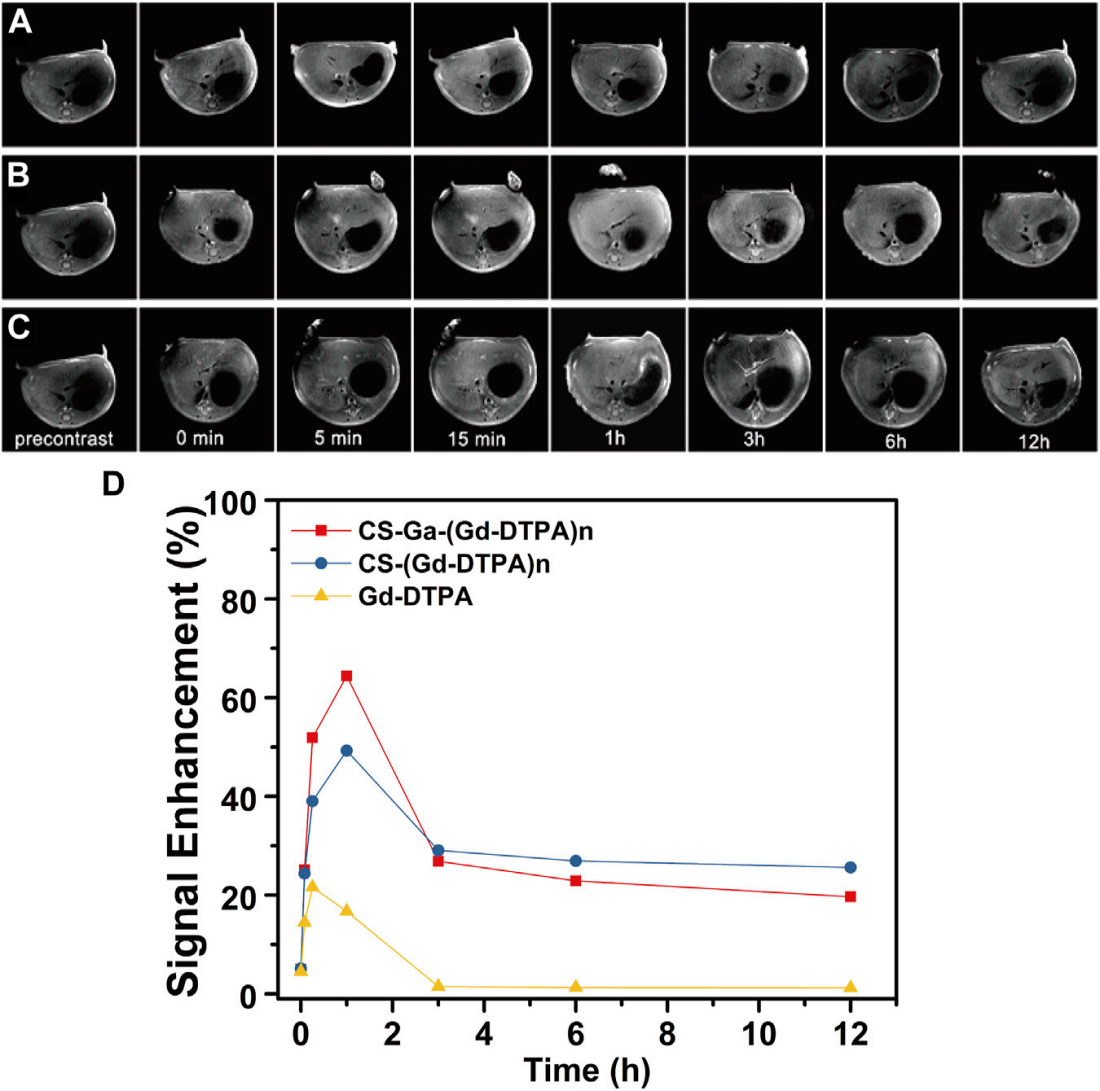
T_1_-weighted MR images of the rats’ liver at precontrast and 0 min, 5 min, 15 min, 1 h, 3 h, 6 h and 12 h after intravenous injection of Gd-DTPA **(A)**, CS-Ga-(Gd-DTPA)_n_ **(B)** and CS-(Gd-DTPA)_n_ **(C)** at dose of 0.03 mM Gd/kg. **(D)** The relative signal enhancement ratio of CS-Ga-(Gd-DTPA)_n,_ CS-(Gd-DTPA)_n_ and Gd-DTPA in rats’ liver calculated by T_1_-weighted MR images.

Sprague-Dawley rats weighting 180–200 g were used to study the MRI signal enhancement of CS-Ga-(Gd-DTPA)_n_ *in vivo*. Figure 5A showed the 2D transverse T_1_ weighted MR images of rats’ liver before and at serial time points after the injection of CS-Ga-(Gd-DTPA)_n,_ and CS-(Gd-DTPA)_n_ and Gd-DTPA at dose of 0.03 mM Gd/kg. The liver signal intensity of CS-Ga-(Gd-DTPA)_n_ and CS-(Gd-DTPA)_n_ treated rats quickly increased and peaked at 1 h post-injection, while that of Gd-DTPA peaked at 15 min post-injection. The relative signal enhancement ratio of CS-Ga-(Gd-DTPA)_n_ and CS-(Gd-DTPA)_n_ peaked at 1 h post-injection (65% and 48%), then decreased remarkably to 30% in 2 h, finally reduced slowly to 25% at 12 h post-injection (Figure 5B). The result indicated that both agents could enhance the MR signal largely and provide a long imaging window. Meanwhile, the relative signal enhancement ratio of Gd-DTPA in liver peaked at 20% at 15 min post-injection, then reduced rapidly to 0% at 3 h post-injection, which was consistent with the previous reports that Gd-DTPA could be excreted quickly in urine from the body as a small molecular MRI contrast agent. In comparison with CS-(Gd-DTPA)_n_, CS-Ga-(Gd-DTPA)_n_ showed higher relative signal enhancement ratio in liver, suggesting that CS-Ga-(Gd-DTPA)_n_ was liver-specific.

### 3.6 *In vivo* biosafety

**FIGURE 6:**
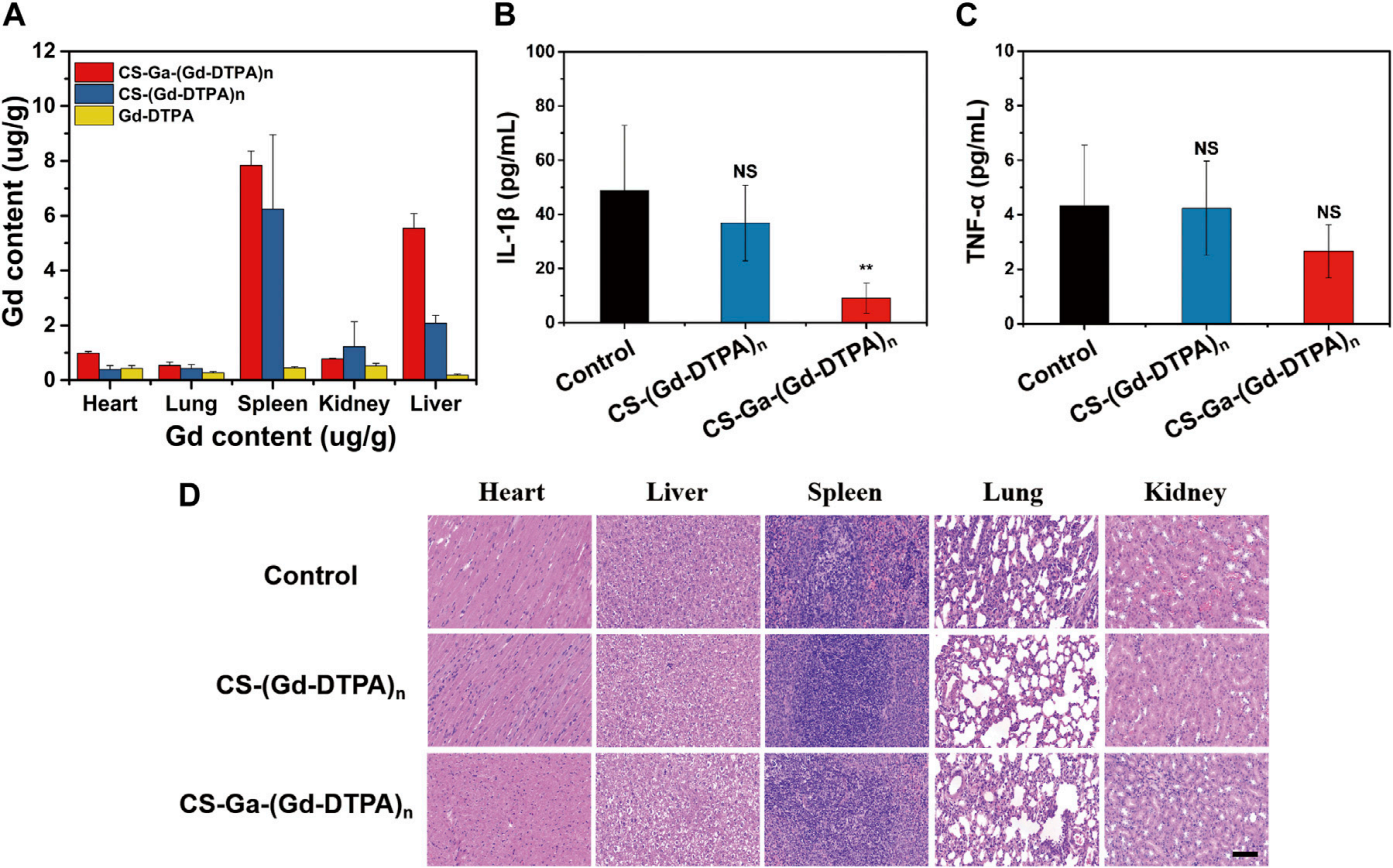
**(A)** Biodistribution of gadolinium (Ⅲ) in rats at 10 days after intravenous injection of CS-Ga-(Gd-DTPA)_n_ and CS-(Gd-DTPA)_n_ at equal dose of 0.03 mM Gd/Kg. **(B,C)** The quantification of IL-1β and TNF-α in the serum of rats at 10 days after intravenous injection of CS-Ga-(Gd-DTPA)_n_ and CS-(Gd DTPA)_n_ at equal dose of 0.03 mM Gd/Kg. NS denotes *p* > 0.05, **p < 0.01 (two tail Student’s t-test for comparison with control). **(D)** HE-staining slices of orgrans (heart, liver, spleen, lung and kidneys) in rats at 10 days after intravenous administration of CS-Ga-(Gd-DTPA)_n_, CS-(Gd-DTPA)_n_ at equal dose of 0.03 mM Gd/Kg, scale bar: 100 μm.

Sprague-Dawley rats weighting 180–200 g were used to evaluate the biodistribution of CS-Ga-(Gd-DTPA)_n_ and CS-(Gd-DTPA)_n_ *in vivo*. The retention of Gd in heart, lung, liver, spleen, kidneys was measured by ICP at 10 days after injection with CS-Ga-(Gd-DTPA)_n_ and CS-(Gd-DTPA)_n_ at a dose of 0.03 mM Gd/kg. The Gd contents in organs of control rats were used as negative control to reduce the interference of instrument error. As shown in Figure 6A, the accumulation of Gd (Ⅲ) in all organs was very low, ranging from 0 to 7.8 ug/g. For both agents, there was nearly no Gd (Ⅲ) retention in heart and lung, a little retention in kidneys, and relatively higher Gd (Ⅲ) retention in spleen and liver. The accumulation of Gd (Ⅲ) in liver of CS-Ga-(Gd-DTPA)_n_ treated rats was relatively higher than that of CS-(Gd-DTPA)_n_ treated rats_,_ which was in accordance with its superior MRI signal enhancement in liver, confirming that CS-Ga-(Gd-DTPA)_n_ was liver specific.

**FIGURE 7:**
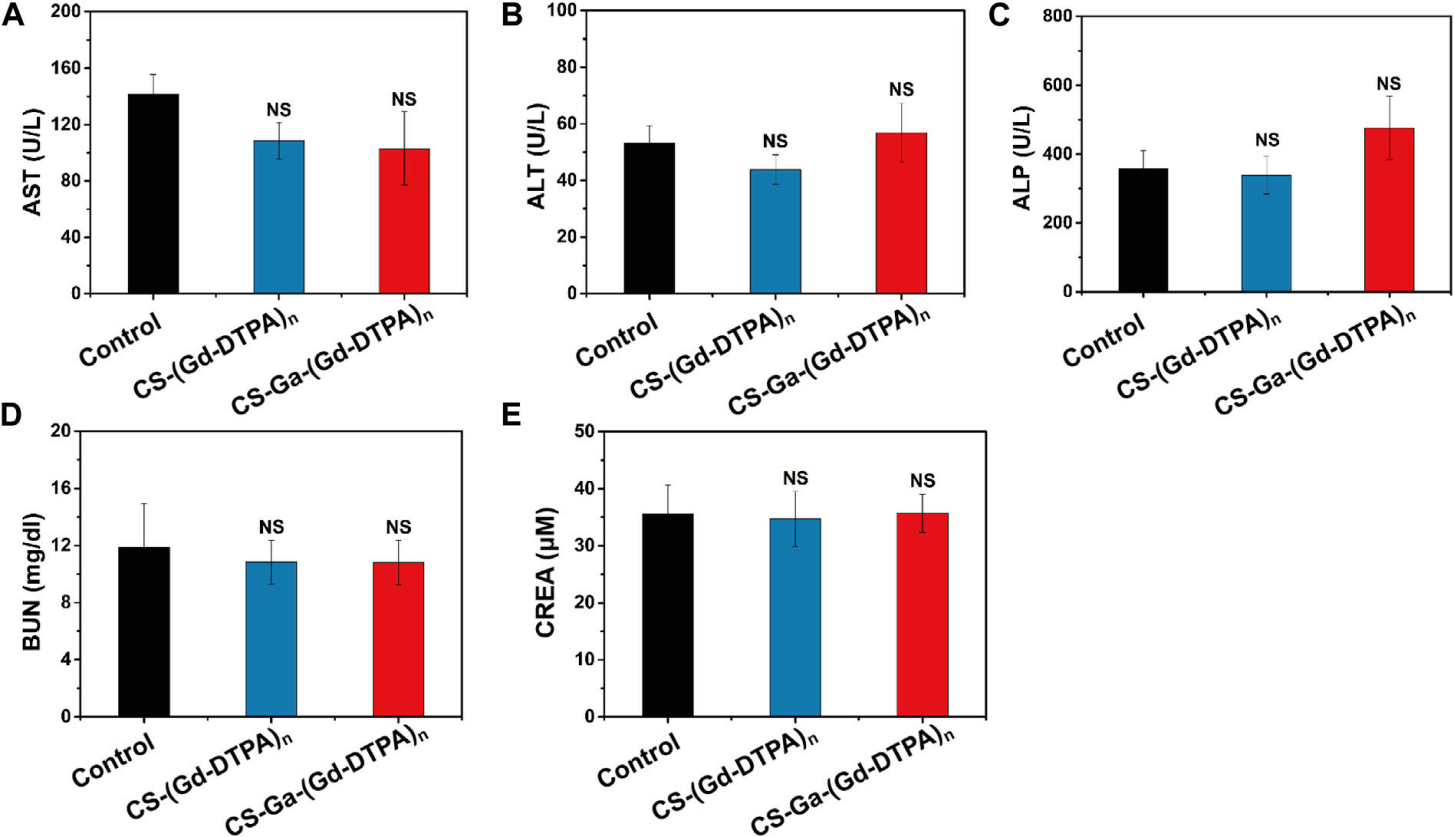
The serum level of AST **(A)**, ALT **(B)**, ALP **(C)**, BUN **(D)** and CREA **(E)** in rats at 10 days after intravenous administration of CS-Ga-(Gd-DTPA)_n_ and CS-(Gd-DTPA)_n_ at equal dose of 0.03 mM Gd/Kg. Data presented as mean ± SD (n = 3). NS denotes *p* > 0.05, (two tail Student’s t-test for comparison with control).

The behavior of all treated rats were normal in 10 days post-injection, implying that CS-Ga-(Gd-DTPA)_n_ and CS-(Gd-DTPA)_n_ showed no acute toxicity on rats. Moreover, there is no increase in the level of IL-1β and TNF-α in serum of rats post-injection of CS-Ga-(Gd-DTPA)_n_ and CS-(Gd-DTPA)_n_ (Figures 6B, C), indicating systemic injection of CS-Ga-(Gd-DTPA)_n_ and CS-(Gd-DTPA)_n_ dosen’t cause inflammation in the whole body. The adverse effects of CS-Ga-(Gd-DTPA)_n_ and CS-(Gd-DTPA)_n_ on organs were further examined by histopathological analysis. The HE-staining of heart, spleen, lung and kidneys tissues showed no damage and abnormal lesions (Figure 6D). Liver is the predominant place in which drugs accumulated and were transformed *in vivo*. As shown in Figure 6D, a certain proportion of vacuolation of hepatocytes was observed in the liver tissue of CS-Ga-(Gd-DTPA)_n_ and CS-(Gd-DTPA)_n_ treated rats, indicating their little effect on liver. However, compared to the control rats, no significant changes is observed in the serum level of aspartate aminotransferase (AST), alanine aminotransferase (ALT), alkaline phosphatase (ALP), blood urea nitrogen (BUN) and creatinine (CREA) (Figure 7), indicating that systemic treatment of CS-Ga-(Gd-DTPA)_n_ and CS-(Gd-DTPA)_n_ does not damage liver and kidney functions. All these data demonstrated CS-Ga-(Gd-DTPA)_n_ and CS-(Gd-DTPA)_n_ have good biocompatibility *in vivo* at the dosage used in our study.

## 4 Conclusion

Compared to Gd-DTPA and CS-(Gd-DTPA)_n_, CS-Ga-(Gd-DTPA)_n_ showed higher relaxivity *in vivo*, remarkable liver targeted performance, and better MRI signal enhancement in liver at a low dose of 0.03 mM Gd/kg. Furthermore, CS-Ga-(Gd-DTPA)_n_ showed good cell and blood compatibility. As macromolecular contrast agent, CS-Ga-(Gd-DTPA)_n_ offered a wider window for clinic examinations and may excrete clearly at 10 days after injection. Furthermore, CS-Ga-(Gd-DTPA)_n_ has no acute toxicity on rats and good biocompatibility *in vivo*. These data together demonstrated that with excellent MRI enhancement property and good biocompatibility, CS-Ga-(Gd-DTPA)_n_ will be a potential candidate for MRI contrast agents.

## Data Availability

The raw data supporting the conclusions of this article will be made available by the authors, without undue reservation.

## References

[B1] Abd Ei-Hack M. E. El-Saadony M. T. Shafi M. E. Zabermawi N. M. Arif M. Batiha G. E. (2020). Antimicrobial and antioxidant properties of chitosan and its derivatives and their applications: A review. Int. J. Biol. Macromol. 164, 2726–2744. 10.1016/j.ijbiomac.2020.08.153 32841671

[B2] Anwanwan D. Singh S. K. Singh S. Saikam V. Singh R. (2020). Challenges in liver cancer and possible treatment approaches. Biochim. Biophys. Acta. Rev. Cancer. 1873 (1), 188314. 10.1016/j.bbcan.2019.188314 31682895 PMC6981221

[B3] Besa C. Lewis S. Pandharipande P. V. Chhatwal J. Kamath A. Cooper N. (2017). Hepatocellular carcinoma detection: Diagnostic performance of a simulated abbreviated MRI protocol combining diffusion-weighted and T1-weighted imaging at the delayed phase post gadoxetic acid. Abdom. Radiol. (NY) 42 (1), 179–190. 10.1007/s00261-016-0841-5 27448609

[B4] Choi J. Y. Lee J. M. Sirlin C. B. (2014). CT and MR imaging diagnosis and staging of hepatocellular carcinoma: Part I. Development, growth, and spread: Key pathologic and imaging aspects. Radiology 272 (3), 635–654. 10.1148/radiol.14132361 25153274 PMC4263631

[B5] Dash M. Chiellini F. Ottenbrite R. M. Chiellini E. (2011). Chitosan—a versatile semi-synthetic polymer in biomedical applications. Prog. Polym. Sci. 36 (8), 981–1014. 10.1016/j.progpolymsci.2011.02.001

[B6] Faletti R. Cassinis M. C. Fonio P. Bergamasco L. Pavan L. J. Rapellino A. (2015). Multiparametric Gd-EOB-DTPA magnetic resonance in diagnosis of HCC: Dynamic study, hepatobiliary phase, and diffusion-weighted imaging compared to histology after orthotopic liver transplantation. Abdom. Imaging 40 (1), 46–55. 10.1007/s00261-014-0180-3 24965896

[B7] Fatima A. Ahmad M. W. Al Saidi A. K. A. Choudhury A. Chang Y. Lee G. H. (2021). Recent advances in gadolinium based contrast agents for bioimaging applications. Nanomater. (Basel) 11 (9), 2449. 10.3390/nano11092449 PMC8465722 34578765

[B8] Forner A. Reig M. Bruix J. (2018). Hepatocellular carcinoma. Lancet 391 (10127), 1301–1314. 10.1016/S0140-6736(18)30010-2 29307467

[B9] Frigaard J. Jensen J. L. Galtung H. K. Hiorth M. (2022). The potential of chitosan in nanomedicine: An overview of the cytotoxicity of chitosan based nanoparticles. Front. Pharmacol. 13, 880377. 10.3389/fphar.2022.880377 35600854 PMC9115560

[B10] Ganesan P. Kulik L. M. (2023). Hepatocellular carcinoma: New developments. Clin. Liver Dis. 27 (1), 85–102. 10.1016/j.cld.2022.08.004 36400469

[B11] Geraldes C. F. G. C. Peters J. A. (2022). MRI contrast agents in glycobiology. Molecules 27 (23), 8297. 10.3390/molecules27238297 36500389 PMC9735696

[B12] Glockner J. F. Lee C. U. Mounajjed T. (2018). Inflammatory hepatic adenomas: Characterization with hepatobiliary MRI contrast agents. Magn. Reson Imaging 47, 103–110. 10.1016/j.mri.2017.12.006 29221964

[B13] Goodwin M. D. Dobson J. E. Sirlin C. B. Lim B. G. Stella D. L. (2011). Diagnostic challenges and pitfalls in MR imaging with hepatocyte-specific contrast agents. Radiographics 31 (6), 1547–1568. 10.1148/rg.316115528 21997981

[B14] Guglielmo F. F. Kania L. M. Ahmad H. M. Roth C. G. Mitchell D. G. (2016). Interpreting body MRI cases: What you need to know to get started. Abdom. Radiol.NY) 41 (11), 2248–2269. 10.1007/s00261-016-0829-1 27444784

[B15] Hata S. Ishii K. (1998). Effect of galactose on binding and endocytosis of asiaioglycoprotein in cultured rat hepatocytes. Ann. Nucl. Med. 12 (5), 255–259. 10.1007/BF03164910 9839486

[B16] Hope T. A. Fowler K. J. Sirlin C. B. Costa E. A. Yee J. Yeh B. M. (2015). Hepatobiliary agents and their role in LI-RADS. Abdom. Imaging 40 (3), 613–625. 10.1007/s00261-014-0227-5 25287679

[B17] Kang T. W. Kong S. Y. Kang D. Kang M. W. Kim Y. K. Kim S. H. (2020). Use of gadoxetic acid-enhanced liver MRI and mortality in more than 30 000 patients with hepatocellular carcinoma: A nationwide analysis. Radiology 295 (1), 114–124. 10.1148/radiol.2020190639 32013789

[B18] Kim M. J. (2020). Improving survival with gadoxetic acid-enhanced MRI for hepatocellular carcinoma. Radiology 295 (1), 125–126. 10.1148/radiol.2020192713 32031470

[B19] Kim S. Y. Park S. H. Wu E. H. Wang Z. J. Hope T. A. Chang W. C. (2015). Transient respiratory motion artifact during arterial phase MRI with gadoxetate disodium: Risk factor analyses. AJR Am. J. Roentgenol. 204 (6), 1220–1227. 10.2214/AJR.14.13677 26001231

[B20] Kyzas G. Z. Bikiaris D. N. (2015). Recent modifications of chitosan for adsorption applications: A critical and systematic review. Mar. Drugs 13 (1), 312–337. 10.3390/md13010312 25584681 PMC4306939

[B21] Lancelot E. Raynaud J. S. Desché P. (2020). Current and future MR contrast agents: Seeking a better chemical stability and relaxivity for optimal safety and efficacy. Invest. Radiol. 55 (9), 578–588. 10.1097/RLI.0000000000000684 32776767

[B22] Lang X. Wang T. Sun M. Chen X. Liu Y. (2020). Advances and applications of chitosan-based nanomaterials as oral delivery carriers: A review. Int. J. Biol. Macromol. 154, 433–445. 10.1016/j.ijbiomac.2020.03.148 32194103

[B23] Lee N. K. Kim S. Kim G. H. Heo J. Seo H. I. Kim T. U. (2012). Significance of the "delayed hyperintense portal vein sign" in the hepatobiliary phase MRI obtained with Gd-EOB-DTPA. J. Magn. Reson. Imaging 36 (3), 678–685. 10.1002/jmri.23700 22649000

[B24] Li H. Meade T. J. (2019). Molecular magnetic resonance imaging with Gd(III)-based contrast agents: Challenges and key advances. J. Am. Chem. Soc. 141 (43), 17025–17041. 10.1021/jacs.9b09149 31593630 PMC6821590

[B25] Lima B. V. Oliveira M. J. Barbosa M. A. Gonçalves R. M. Castro F. (2021). Immunomodulatory potential of chitosan-based materials for cancer therapy: A systematic review of *in vitro*, *in vivo* and clinical studies. Biomater. Sci. 9 (9), 3209–3227. 10.1039/d0bm01984d 33949372

[B26] Llovet J. M. Kelley R. K. Villanueva A. Singal A. G. Pikarsky E. Roayaie S. (2021). Hepatocellular carcinoma. Nat. Rev. Dis. Prim. 7 (1), 6. 10.1038/s41572-020-00240-3 33479224 PMC13537433

[B27] Maluccio M. Covey A. (2012). Recent progress in understanding, diagnosing, and treating hepatocellular carcinoma. CA Cancer J. Clin. 62 (6), 394–399. 10.3322/caac.21161 23070690

[B28] McDonald J. S. McDonald R. J. (2020). MR imaging safety considerations of gadolinium-based contrast agents: Gadolinium retention and nephrogenic systemic fibrosis. Magn. Reson. Imaging Clin. N. Am. 28 (4), 497–507. 10.1016/j.mric.2020.06.001 33040991

[B29] McInnes M. D. Hibbert R. M. Inácio J. R. Schieda N. (2015). Focal nodular hyperplasia and hepatocellular adenoma: Accuracy of gadoxetic acid-enhanced MR imaging-a systematic review. Radiology 277 (3), 927. 10.1148/radiol.2015154033 26599935

[B30] Mukthavaram R. Marepally S. Venkata M. Y. Vegi G. N. Sistla R. Chaudhuri A. (2009). Cationic glycolipids with cyclic and open galactose head groups for the selective targeting of genes to mouse liver. Biomaterials 30 (12), 2369–2384. 10.1016/j.biomaterials.2008.12.074 19157538

[B31] Muxika A. Etxabide A. Uranga J. Guerrero P. de la Caba K. (2017). Chitosan as a bioactive polymer: Processing, properties and applications. Int. J. Biol. Macromol. 105 (2), 1358–1368. 10.1016/j.ijbiomac.2017.07.087 28735006

[B32] Okada M. Murakami T. Kuwatsuru R. Nakamura Y. Isoda H. Goshima S. (2016). Biochemical and clinical predictive approach and time point analysis of hepatobiliary phase liver enhancement on Gd-EOB-DTPA-enhanced MR images: A multicenter study. Radiology 281 (2), 474–483. 10.1148/radiol.2016151061 27195437

[B33] Oluwasola I. E. Ahmad A. L. Shoparwe N. F. Ismail S. (2022). Gadolinium based contrast agents (GBCAs): Uniqueness, aquatic toxicity concerns, and prospective remediation. J. Contam. Hydrol. 250, 104057. 10.1016/j.jconhyd.2022.104057 36130428

[B34] Sung H. Ferlay J. Siegel R. L. Laversanne M. Soerjomataram I. Jemal A. (2021). Global cancer statistics 2020: GLOBOCAN estimates of incidence and mortality worldwide for 36 cancers in 185 countries. Ca. Cancer J. Clin. 71 (3), 209–249. 10.3322/caac.21660 33538338

[B35] Vilgrain V. Van Beers B. E. Pastor C. M. (2016). Insights into the diagnosis of hepatocellular carcinomas with hepatobiliary MRI. J. Hepatol. 64 (3), 708–716. 10.1016/j.jhep.2015.11.016 26632635

[B36] Villanueva A. (2019). Hepatocellular carcinoma. N. Engl. J. Med. 380 (15), 1450–1462. 10.1056/NEJMra1713263 30970190

[B40] Wang X. Xu L. Ren Z. Fan M. Zhang J. Qi H. (2019). A novel manganese chelated macromolecular MRI contrast agent based on O-carboxymethyl chitosan derivatives. Colloids Surf. B: Biointerfaces 183, 110452. 10.1016/j.colsurfb.2019.110452 31473409

[B37] Welle C. L. Guglielmo F. F. Venkatesh S. K. (2020). MRI of the liver: Choosing the right contrast agent. Abdom. Radiol. (NY) 45 (2), 384–392. 10.1007/s00261-019-02162-5 31392396

[B38] Zhang H. Guo Y. Jiao J. Qiu Y. Miao Y. He Y. (2022). A hepatocyte-targeting nanoparticle for enhanced hepatobiliary magnetic resonance imaging. Nat. Biomed. Eng. 7, 221–235. 10.1038/s41551-022-00975-2 36536254

[B39] Zheng Z. Pan X. Xu J. Wu Z. Zhang Y. Wang K. (2020). Advances in tracking of polysaccharides *in vivo*: Labeling strategies, potential factors and applications based on pharmacokinetic characteristics. Int. J. Biol. Macromol. 163, 1403–1420. 10.1016/j.ijbiomac.2020.07.210 32738323

